# Effectiveness of experimental composites containing antimicrobial agents for orthodontic applications

**DOI:** 10.1007/s10266-026-01319-1

**Published:** 2026-02-03

**Authors:** Caroline Veiga Malavasi, Eduardo Moreira da Silva, Flávia Pires Rodrigues, José Guilherme Antunes Guimarães, Maristela Barbosa Portela, Guilherme Goulart Cabral-Oliveira, Laiza Tatiana Poskus

**Affiliations:** 1 School of Dentistry, Labiom-R, Universidade Federal Fluminense, Niterói, RJ Brazil; 2 School of Dentistry, Oral Biology Division, University of Leeds, Leeds, England; 3 School of Medical Sciences, Department of Microbiology, Universidade Do Estado Do Rio de Janeiro, Rio de Janeiro, RJ Brazil

**Keywords:** Antimicrobial agents, CFU, Enamel hardness, Composites, Shear bond strength

## Abstract

This study evaluated the effectiveness of experimental composites containing antimicrobial agents such as nisin (N) and stannous fluoride (SnF_2_) against *S. mutans*, for orthodontic applications. First, the antimicrobial agents were added at varying concentrations (%) into a base material consisting of methacrylate monomers and inorganic particles, forming the groups: control (C), N4% (N4), N7% (N7), N10% (N10), SnF_2_0.6% (SnF_2_0.6), SnF_2_1.5% (SnF_2_1.5), SnF_2_ 3% (SnF_2_3), and N4% + SnF_2_ 0. 6% (N4SnF_2_0.6). The degree of conversion (DC%) and colony-forming units (CFU) were evaluated. Brackets were bonded onto the enamel of bovine incisors using these composites, and the enamel hardness was measured at distances of 500, 550, and 600 µm from the bracket, before and after the cariogenic challenge. The shear bond strength was also investigated, and the composite remnant on the enamel was assessed using the ARI score. Data were analyzed using analysis of variance and Tukey’s test (α = 0.05). Of all the groups, only N10 led to lower DC% (*p* < 0.05). CFU was significantly reduced for all groups when compared to C (*p* < 0.05), with SnF_2_ showing the highest inhibition for all concentrations. After the cariogenic challenge, for all distances from the brackets, the decrease in hardness was lower for all experimental composites than for C (*p* < 0.05). SBS was lower only for the N4SnF_2_0.6 when compared to C. Adhesive failure was more frequent, with the composite remnant covering less than 50% of the adhesive area. In conclusion, adding those antimicrobial agents effectively inhibited *S. mutans* and protected enamel around brackets without compromising most mechanical properties. However, the highest nisin concentration (10%) adversely affected the degree of conversion, and the combination of nisin and stannous fluoride reduced bond strength.

## Introduction

Enamel demineralization around orthodontic accessories remains a significant problem for patients with fixed appliances [1]. Organic acids from the biofilm colonized by cariogenic microbiota can induce enamel demineralization, leading to a high prevalence of dental white spot lesions or caries formation [2], particularly around brackets [3], which can develop in as little as 3 months. [3, 4]. While patients’ oral health habits, including oral hygiene and diet control, are crucial, developing dental materials to prevent demineralization is also essential.

Resin materials with antimicrobial potential have been extensively investigated, including adhesive systems [5], composites [6], and cements [7]. Although additives such as chlorhexidine [8], metallic particles (silver, zinc, and others) [8], catechin [9], and coffee compounds [6] have demonstrated antimicrobial activity, their release into the oral environment may compromise their long-term anti-caries efficacy [6, 8]. Conversely, immobilized antimicrobial agents in the polymer chain through copolymerization, such as quaternary ammonium monomers (QAM) and methacryloyloxy-dodecylpyridinium bromide (MDPB), can offer prolonged antimicrobial action by contact [8]. However, their effectiveness can be hindered by the formation of a salivary protein layer that prevents bacterial contact. [8].

Incorporating new antimicrobial agents in resin materials seems to be a relevant strategy to prevent dental demineralization in surrounding areas. Nisin, a natural peptide produced by Lactococcus lactis, has shown efficacy against oral bacteria such as *Streptococcus mutans* (*S. mutans*) [10]. This is due to its mechanism of forming pores in the bacterial cell membrane, as a result of its interaction with anionic lipids. These pores allow an immediate exit of small cytoplasmic components such as adenosine triphosphate (ATP), amino acids, and rubidium aggregates from cells, and the collapse of vital ion gradients, leading to cell death [11, 12]. At the same time, potential cytotoxicity was not observed in oral cells [13], suggesting that it could be used to prevent dental caries [10]. When incorporated into dental adhesives, this substance was able to inhibit *S. Mutans* [14], as well as multispecies biofilms [15], without affecting their adhesive properties [14, 15]. This inhibition is not only present in dental microbiota, but also within a broad-spectrum action against Gram-positive bacteria, widely used for food preservation [16]. However, differences in the growth of *S. mutans* could not be detected in a previous study [17], when nisin and monocalcium phosphate monohydrate (MCPM) were added concomitantly in different concentrations to orthodontic adhesives.

Considering other antimicrobial agents, fluorides are well recognized for their anti-caries action and are commonly present in oral care products like toothpastes, mouthwashes, and water [18]. Despite the widespread use of sodium fluoride (NaF), studies have shown that stannous fluoride (SnF_2_) exhibits greater efficacy in inhibiting *S. mutans* and preventing the formation of caries lesions [19]. In addition to the formation of a more effective acid-resistant layer on the tooth surface with a decrease in mineral loss [20], the association of fluor and stannous ions is also able to reduce the acidogenicity of dental plaque [21], disrupting the transport system of bacterial membranes, and inhibiting essential enzyme systems involved in sugar fermentation [22].

Thus, considering the need for the development of new materials that can inhibit caries formation around orthodontic appliances, the scarce literature about the antimicrobial action of nisin and stannous fluoride added to polymeric materials for orthodontic applications, and the contradictory results found in the literature in this regard for nisin, this study aimed to develop experimental composites for orthodontic applications combining nisin and stannous fluoride to verify: (i) their antimicrobial effect against *S. mutans*; (ii) to investigate the enamel hardness around orthodontic appliances; and (iii) the bond strength between the brackets and the enamel structure. The null hypothesis of the present study was that the antimicrobial effect against *S. mutans*, the enamel hardness around orthodontic appliances, and the bond strength between the brackets and the enamel structure would not be influenced by the different concentrations of nisin, stannous fluoride, or their combination in the experimental composites used for fixing orthodontic appliances.

## Materials and methods

### Formulation of the experimental composites

A mixture of 60% wt. of bisphenol-A-glycidyl dimethacrylate (Bis-GMA) and 40% wt. of triethylene glycol dimethacrylate (TEGDMA) (Esstech Inc., Essington, PA, USA) with 1% camphorquinone and 1% ethyl N, N-dimethyl-4aminobenzoate (EDMAB amine) (Sigma-Aldrich, St. Louis, MO, USA) was obtained at room temperature. Pre-silanized silica with an average size of 7 nm and SiO2BaO particles with an average size of 0.7 µm (Esstech Inc., Essington, PA, USA) were added (1:9 w/w) to the resin matrix up to 65wt% of filler content. Then the antimicrobial agents nisin (Sigma-Aldrich, St. Louis, MO, USA) and stannous fluoride (Sigma-Aldrich, St. Louis, MO, USA) were added according to Table 1, forming seven experimental groups plus the control.

**Table 1:** Experimental groups

Groups	Antimicrobial agent (wt%)
Control	0
N4	Nisin 4%
N7	Nisin 7%
N10	Nisin 10%
SnF_2_0.6	Stannous fluoride 0.6%
SnF_2_1.5	Stannous fluoride 1.5%
SnF_2_3	Stannous fluoride 3%
N4SnF_2_0.6	Nisin 4% + stannous fluoride 0.6%

### Sample size calculation

Based on the values reported in previous studies [14, 23], the effect size (d) was calculated as 2.056 for degree of conversion, 6.708 for CFU, 3.083 for microhardness, and 1.226 for microshear bond strength. The power of the study was set at 80% with a significance level of α = 0.05, and the minimum sample size per group was calculated. Considering this, the authors decided to include 5, 3, 5, and 15 specimens per group for each test, respectively.

### Degree of conversion (GC%)

The degree of monomeric conversion (*n* = 5) was evaluated by Fourier transform infrared spectroscopy (FTIR) using the attenuated total reflectance technique—ATR—(Alpha-P / Platinum ATR Module, Bruker Optics GmbH, Ettlingen, Germany). A small increment of each composite was placed onto the FTIR spectrometer’s ATR crystal, and the spectra were recorded between 1.500 and 1.800 cm^-1^, using 40 scans at a resolution of 4 cm^-1^. The composites were then light-cured for 20 s (LED Demi, Kerr, Wisconsin/USA, irradiance 800 mW/cm2), and the spectra were re-recorded. The GC% was calculated as the ratio between the integrals of the absorption bands of the aliphatic C = C bond (1638 cm^-1^) and the C = O bond (1608 cm^-1^), used as an internal standard, which was obtained from the cured and uncured mixtures, using the following formula:

1 $$GC\%=\left(1-\frac{Cured}{Uncured}\right)x 100$$

### Colony-forming units (CFU) count

Resin-composite discs (5 mm Ø X 1 mm thickness) were obtained (*n* = 3) using a custom-made steel mold. The experimental composites were introduced into the mold and covered with a polyester matrix and a glass microscope slide. A mass of 0.5 kg was applied to extrude the excesses. The light-curing was then performed for 40 s (LED Demi, Kerr, Wisconsin/USA, irradiance 800 mW/cm^2^) from both sides, and the excesses were removed. Based on the methodology described by Perez et al. [24], the discs were placed in a 96-well, flat-bottom polystyrene cell culture plate (Costar 3599, Corning Inc., NY, USA). A bacterial suspension of the *S. mutans* strain (ATCC 25175), containing approximately 10^7^ CFU/mL (0.16 OD at 580 nm) in TSB (trypticase soy broth, Becton Dickinson, New Jersey, USA), was also diluted in TSB until an approximate concentration of 10^4^ CFU/mL was obtained. 5-µL of the suspension (approximately 50 CFU) was placed on the surface of the samples and kept in contact for 30 min inside a humid chamber to avoid drying out of the culture medium.

After this period, 200 µL of TSA (liquefied) containing 0.5% yeast extract (Becton Dickinson, New Jersey, USA) was added to each well, and the plate was incubated for 24 h at 37 °C, in the presence of CO_2_. To observe the viability of microorganisms, a control with the inoculum deposited in wells containing only the culture medium (without discs) was used. Sterility control was performed with the deposition of a pure culture medium without the addition of microorganisms or discs. After incubation, the number of colony-forming units was counted using a stereomicroscope with 1000 × magnification (Nikon C-DSD230 Tokyo, Japan). The test was performed in triplicate. The percentage of inhibition was calculated based on the total of CFU initially present on the samples (approximately 50 CFU).

### Enamel surface hardness

**Fig. 1:**
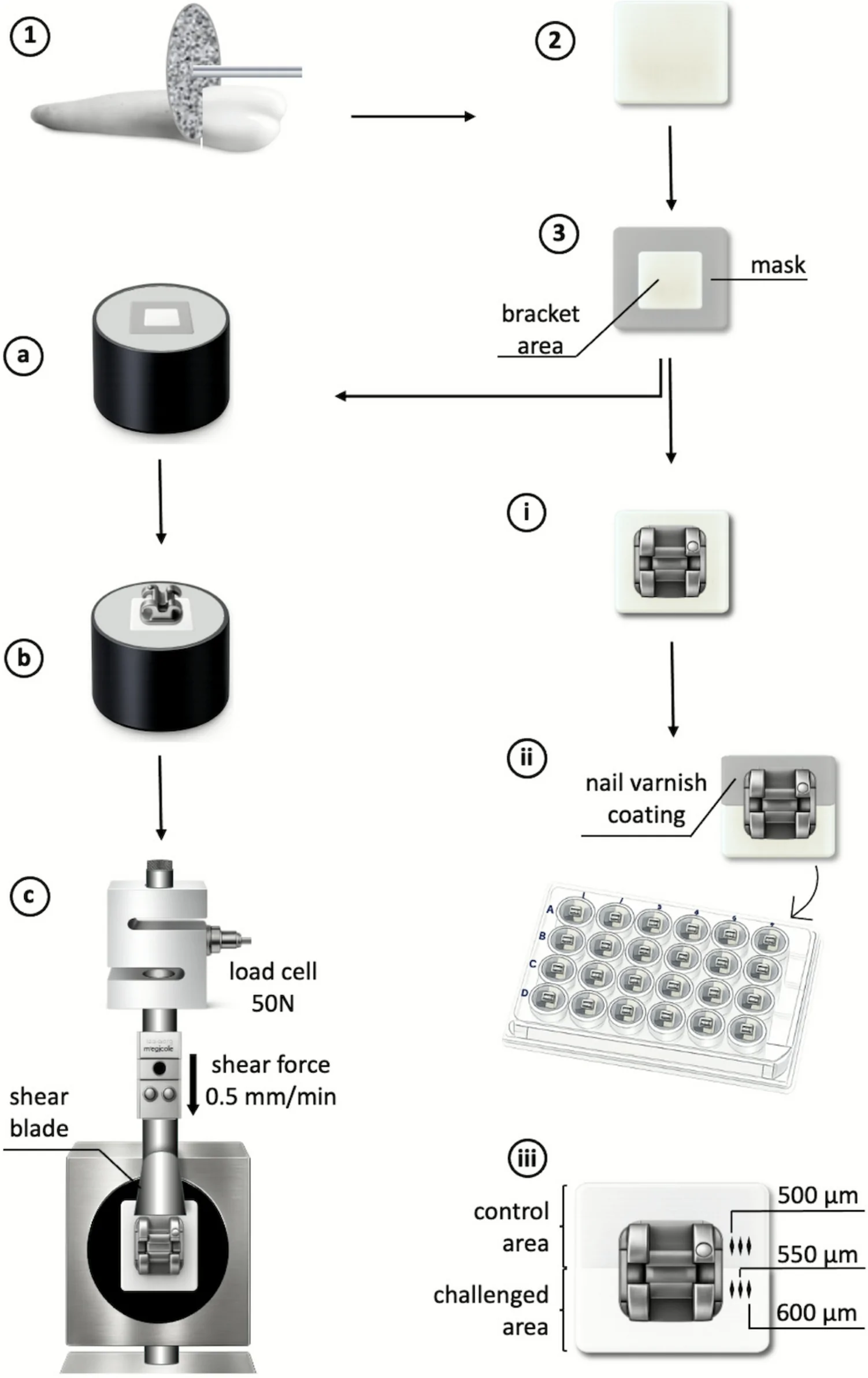
Schematic representation of the shear bond strength and microhardness tests setup: 1) root removing; 2) flattened and polished bovine enamel fragment; 3) delimitation of the bracket cementation area with an adhesive tape mask; **a** embedded enamel fragment; **b** orthodontic bracket cemented on the enamel surface; **c** shear bond strength test (*n* = 15); i) bracket cemented on the enamel surface; ii) coating half of the fragment’s surface with nail varnish for the cariogenic challenge; iii) microhardness test (*n* = 5)

Forty bovine incisors had their buccal enamel surfaces lightly ground using silicon carbide papers (400, 600, and 1200 grit) under constant water cooling and then polished with diamond paste (0.3 µm). After removing the roots, bovine fragments with dimensions of approximately 6 mm × 6 mm × 2 mm were obtained using a cutting machine (IsoMet 1000, Buëhler, Lake Bluff, IL, USA). An adhesive tape mask (3 M Scotch, Campinas, SP, Brazil) was used to protect the enamel beyond the area where the flat stainless-steel bracket (Edgewise Standard; Morelli, Sorocaba, Brazil) would be fixed (Fig. 1). Subsequently, the unprotected enamel area (approximately 3.84 × 3.73 mm) was etched with 37% phosphoric acid for 30 s, rinsed for 60 s, and air-dried for 5 s. One coat of the adhesive (Single Bond, 3 M ESPE, St. Paul, MN, USA) was actively applied on the enamel surface for 10 s, gently air-sprayed for 5 s, and light-cured using an LED unit for 20 s (Demi, Kerr, Wisconsin, USA), at 800 mW/cm^2^. Finally, the experimental composites were placed on the bracket surfaces (n = 5), which were carefully positioned on the bonded enamel surfaces. After removing the excesses, each side of the bracket was light-cured for 20 s, the adhesive tape was removed, and the subjacent enamel surface was cleaned with 70% ethanol and air-dried. Half the buccal surface was covered with two consecutive layers of acid-resistant nail varnish to protect against the cariogenic challenge, serving as the control.

The enamel-bracket assembly was initially disinfected and exposed to ultraviolet light in the laminar flow hood for 30 min on each side, then placed and fixed in 24-well plates [25]. A 2 mL of *S. mutans* cell suspension in brain heart infusion was added to each well (brain heart infusion—BHI, Difco, Sparks, USA), supplemented with 2% sucrose. The mature biofilm was formed from *S. mutans* (ATCC 25175—American Type Culture Collection, Rio de Janeiro, RJ, Brazil) and cultivated for 24 h at 37ºC, under conditions of low oxygen tension.

The bacterial suspension was adjusted to an optical density of 0.5 following the McFarland scale (Biomérieux Brazil AS, RJ, Brazil) at 550 nm using a UV/Vis spectrophotometer (Beckman Coulter DU 530, LifeScience, San Diego, CA, USA). The suspension was diluted to 1:100, and 10 µL of this suspension was added to each well of the culture plates, containing a specimen with 2 mL of BHI broth supplemented with 2% sucrose. The 24-well culture plates were incubated at 37 °C in a condition of low oxygen tension for 48 h, the culture medium being replaced every 24 h.

After the cariogenic challenge, the brackets were detached, and the nail varnish was removed with acetone. Knoop microhardness (Micromet 5104, Full MHT software, Büehler, Lake Bluff, IL, USA) was determined not only on the enamel submitted to the cariogenic challenge (KHNF), but also on the protected enamel (KHNI) at three distances from the bracket area, with a distance of 50 µm between them, and using a 50 g load for 10 s. The first indentation was performed at 500 µm from the bracket to ensure that there would be no resin remaining on the enamel surface. Five indentations were performed at each distance, and an average was calculated. The percentage of the decrease in enamel hardness was also calculated using Eq. (2):

2 $$\Delta \%KHN=(1-\frac{KHNF}{KNHI}) x 100$$

### Shear bond strength (MPa)

One hundred and twenty bovine incisors were prepared as described before, embedded in acrylic resin, and stored in distilled water for 24 h. Then the specimens (*n* = 15) were loaded parallel to the adhesive interface onto a universal testing machine (EMIC DL 2000, São José dos Pinhais, SP, Brazil) at a crosshead speed of 0.5 mm/min until bonding failure (Fig. 1). The shear bond strength (SBS) was calculated based on the load at debonding and the bracket base surface area (3.84 × 3.73 mm), using Eq. (3):

3 $$SBS \left(MPa\right)=\frac{N}{Area}$$

After debonding, the mode of failure was analyzed on the tooth surface at 40 × magnifications using a stereomicroscope (Olympus SZ-40, Japan), and classified considering the ARI (Adhesive remaining index) score described by Artun & Bengland [26]:

Score 0: no composite remained on the tooth surface;

Score 1: less than half the composite remained on the enamel surface;

Score 2: more than half of the composite remained on the enamel surface;

Score 3: all composite remained on the enamel surface.

### Statistical analysis

Data were analyzed using the Statgraphics 5.1 software (Manugistics, Rockville, MD, USA). First, the Shapiro–Wilk and Levene tests were used to determine whether the data were homoscedastic and normally distributed. Then the one-way analysis of variance was performed for the CFU count, degree of conversion, and bond strength assays. The two-way analysis of variance was applied for the hardness test. Tukey’s post hoc test was used at a significance level of 5% for contrast of the means. The Chi-square (*χ* ^2^) test was used to evaluate the failure mode (ARI).

## Results

### Degree of conversion (DC%)

**Fig. 2:**
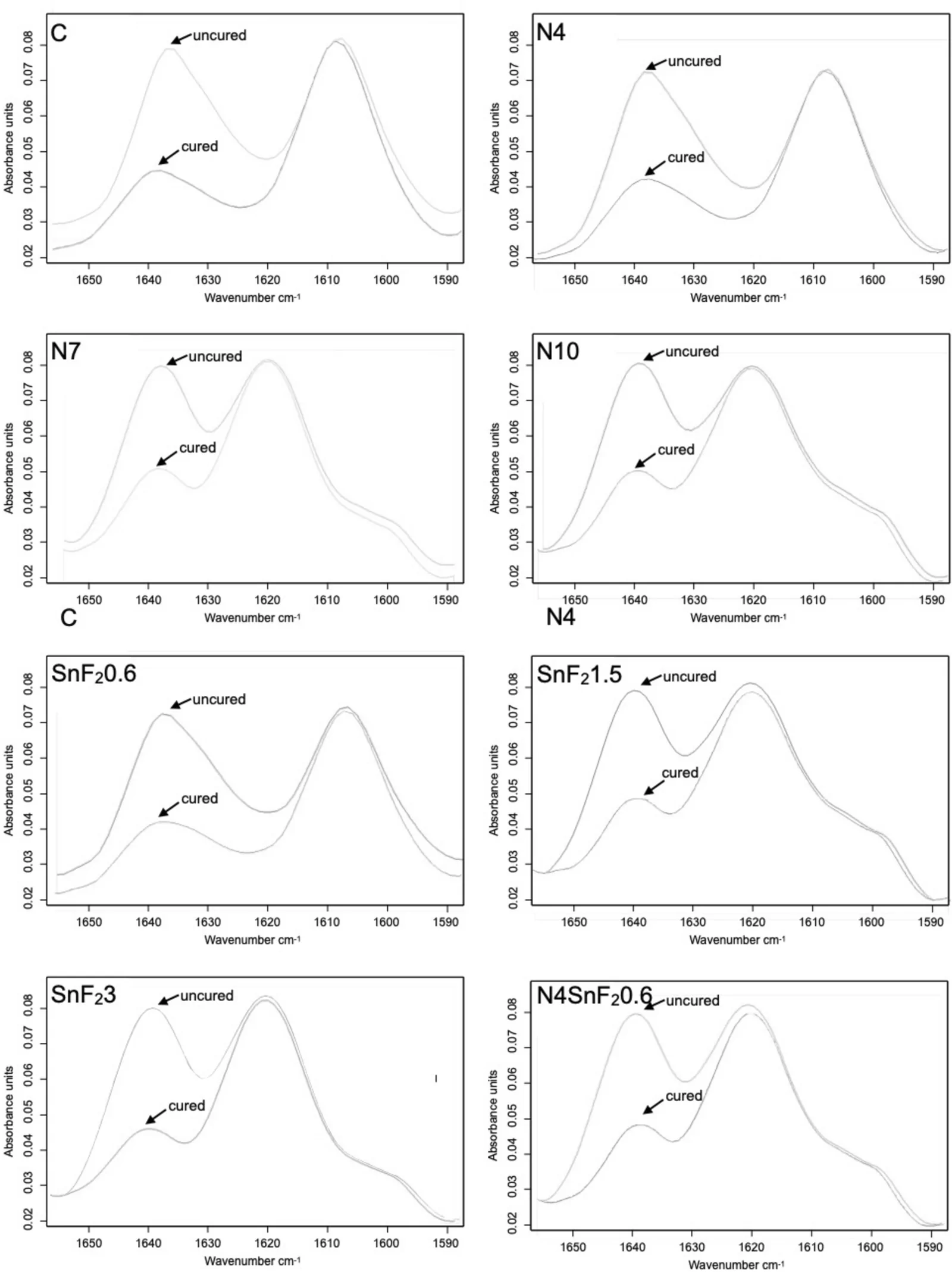
ATR-FTIR spectrum for each experimental group

According to Table 2, only the experimental resin composite containing nisin at 10% showed a lower DC% than the control group (*p* < 0.05). The other groups showed similar (N4 and N7) or higher (Sn_2_F0.6, Sn_2_F1.5, Sn_2_F3, and N4Sn_2_F0.6) DC% than the control group. Figure 2 shows the spectrum for each experimental group, for the cured and uncured samples.

**Table 2:** Means of degree of conversion (DC%) for the experimental groups

Groups	DC% (standard deviation)^*^
Control	64.4 (0.04) a
N4	64 (0.03) a
N7	55.8 (0.06) ab
N10	51.2 (0.06) b
SnF_2_0.6	76.8 (0.04) c
SnF_2_1.5	79.4 (0.07) c
SnF_2_3	81.6 (0.05) c
N4SnF_2_0.6	77.8 (0.07) c

^*^Different letters indicate statistically significant differences

### Colony-forming units (CFU) count

According to Table 3, it can be observed that the addition of nisin and stannous fluoride, either alone or in combination, was able to inhibit the growth of *S. mutans* compared to the control (*p* < 0.05). Stannous fluoride caused more significant inhibition than nisin (*p* < 0.05). The addition of nisin at the highest concentration (10%), or together with stannous fluoride, was more capable of inhibiting *S. mutans* than at its lower concentrations (*p* < 0.05).

**Table 3:** *S. mutans* CFU inhibition (%)

Groups	% Inhibition (standard deviation)^*^
Control	6.6 (0.07) a
N4	66.6 (0.04) b
N7	60.6 (0.04) b
N10	78.6 (0.01) c
SnF_2_0.6	100 (0.00) d
SnF_2_1.5	98.0 (0.03) d
SnF_2_3	96.6 (0.03) d
N4SnF_2_0.6	78.0 (0.2) c

^*^Different letters indicate statistically significant differences

### Enamel surface hardness

According to Table 4, it can be observed that all resin composites containing antimicrobial agents showed a lower decrease in enamel hardness than the control group (*p* < 0.05) at all distances from the brackets. At 600 µm, except for the N4SnF_2_0.6 group, the groups containing antimicrobial agents showed a higher decrease in enamel hardness than those found for the shorter distances, but still lower than the control group.

**Table 4:** Means of decrease in enamel hardness (ΔKHN%) at the different distances from the brackets for the experimental groups

Groups	500 µm ΔKHN% (standard deviation)	550 µm ΔKHN% (standard deviation)	600 µm ΔKHN% (standard deviation)
Control	48.07 (0.05) Aa	44.52 (0.08) Aa	44.57 (0.06) Aa
N4	15.69 (0.03) Ab	25.49 (0.06) ABb	36.79 (0.09) Bb
N7	14.91(0.06) Ab	22.71 (0.06) ABb	33.13 (0.02) Bb
N10	11.84 (0.04) Ab	19.71 (0.05) Ab	32.13 (0.05) Bb
SnF_2_0.6	11.96 (0.05) Ab	23.87 (0.07) Ab	41.21 (0.05) Bb
SnF_2_1.5	6.39 (0.03) Ab	19.02 (0.1) Ab	40.16 (0.1) Bb
SnF_2_3	5.76 (0.04) Ab	11.11 (0.06) Ab	38.02 (0.04) Bb
N4SnF_2_0.6	11.61 (0.03) Ab	21.44 (0.09) Ab	20.81 (0.08) Ac

*Different letters (uppercase horizontally and lowercase vertically) indicate statistically significant differences

### Shear bond strength (SBS)

**Fig. 3:**
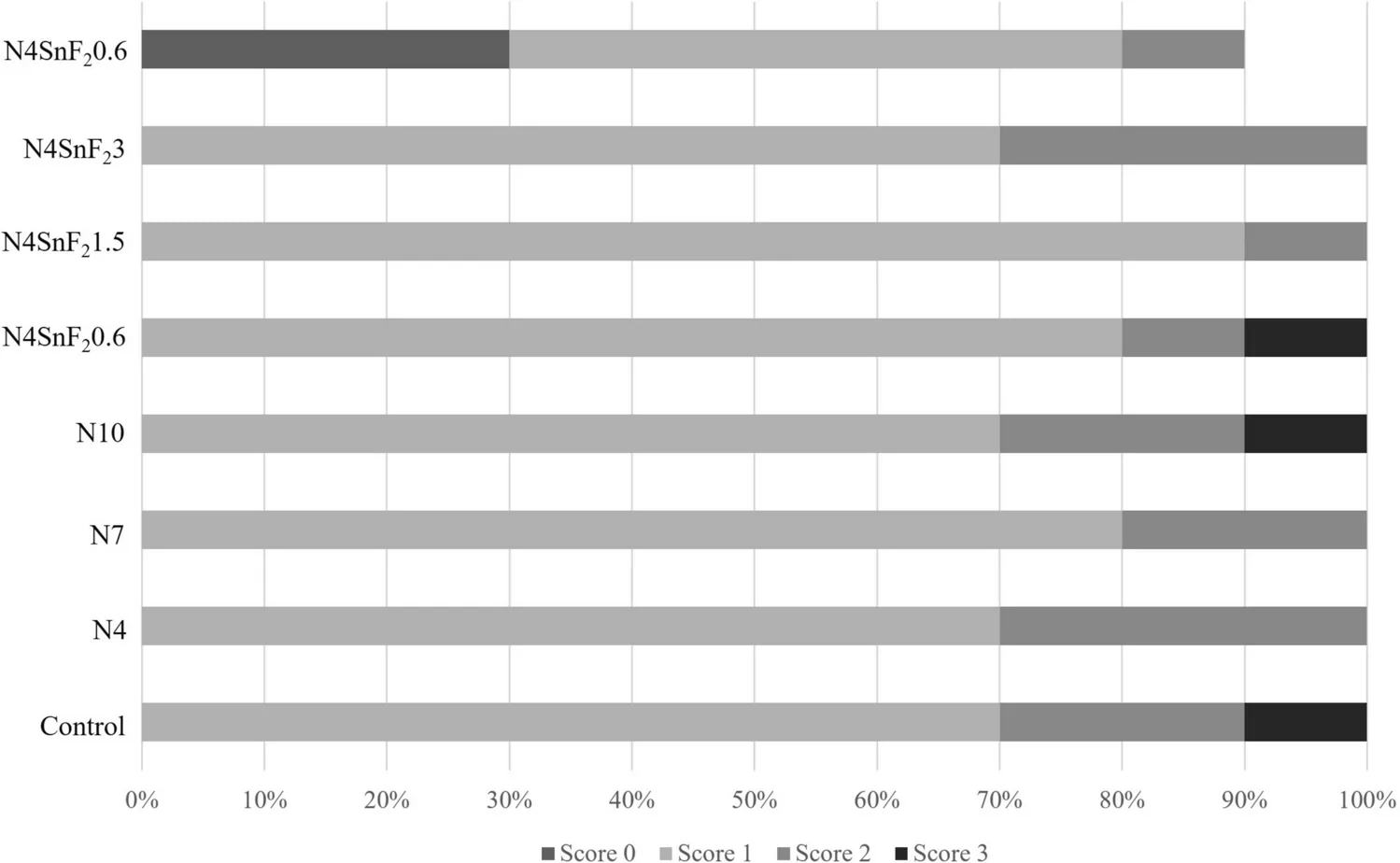
Percentage of ARI score after debonding

According to Table 5, only the N4SnF_2_0.6 group showed lower shear bond strength (*p* < 0.05) than the control group. In contrast, the other groups reached statistically similar SBSs to the control group. The Chi-square (*χ* ^2^) test showed significant differences for the failure mode (*p* < 0.05). The most common ARI score observed for the experimental groups was 1 (Fig. 3), indicating a predominance of adhesive failure. The score 0 was detected only for the N4SnF_2_0.6 group.

**Table 5:** Means of shear bond strength (MPa) for the experimental groups

Groups	Shear bond strength (standard deviation)^*^
Control	25.79 (0.01) a
N4	24.26 (0.03) a
N7	23.75 (0.01) ab
N10	23.81 (0.01) ab
SnF_2_0.6	25.04 (0.01) a
SnF_2_1.5	23.60 (0.01) ab
SnF_2_3	23.60 (0.03) ab
N4SnF_2_0.6	17.30 (0.02) b

*Different letters indicate statistically significant differences

## Discussion

Based on the results of the present study, the null hypothesis was rejected. The authors found that incorporating nisin or stannous fluoride, or both, in composites was capable not only of inhibiting the *S. mutans*, but also of protecting the dental enamel around orthodontic accessories. Notably, there was a significant decrease in the degree of conversion for the group containing the concentration of nisin (10%) (Table 4), thus this concentration would not be recommended, since its higher capacity to inhibit *S. mutans* was not translated into greater enamel protection around the brackets, and could impair the material’s physical properties as shown in previous studies [27]. Also, although the N4 had shown DC% similar to the control group, when stannous fluoride was added (N4SnF_2_0.6), the degree of conversion became higher than that of the control. Indeed, all the other groups containing the salt SnF_2_ showed a higher degree of conversion than the control. Concerning the Knoop hardness, all resin composites containing antimicrobial agents led to a lower decrease in enamel hardness than the control groups at all distances from the brackets, and the SBS was impaired for the N4SnF_2_0.6 group.

Previous studies [28, 29] have shown that the addition of diphenyl iodonium hexafluorophosphate (DPI) into experimental resin materials could improve the DC%. The use of this salt along with a camphorquinone and an amine, both commonly present in dental resin materials, leads to its decomposition, facilitated by the very low energy of the C-I bond from the DPI. Due to light absorbance, camphorquinone can provide an electron that reduces the salt into phenyl radicals. These can react with residual amines, deducting protons and creating new free radicals [30, 31], or with inactive camphorquinone radicals, as an electron acceptor, decreasing the termination rate and enhancing the initiation [31]. So, the higher quantities of free radicals would be responsible for the higher degree of conversion of resin materials. In the present study, the composites with SnF_2_ developed a higher DC% (Table 2). Although the bond between Sn and F has an ionic character, Sn is a post-transition metal that brings a covalent contribution to this bond. When this phenomenon takes place, the electronic cloud of the anion (F^−^, here) may be distorted by the cation (Sn^2+^), which creates a region of greater electron density between them [32]. Thus, we can hypothesize that this covalent contribution could have allowed Sn^2+^ to be reduced by camphorquinone, thereby starting the same reactions produced by the diphenyl iodonium hexafluorophosphate salt, i.e., formation of a high level of free radicals and enhancement of the initiation phase of the polymerization [33].

In the present study, nisin alone inhibited the growth of *S. mutans* (Table 3): the greater the content, the higher the inhibitory effect. This result is supported by the study of Tong et al. [10] which showed that nisin played an effective antimicrobial activity against *S. mutans* and other bacteria. Moreover, although there are no studies in the literature that have incorporated nisin alone into dental composites, when incorporated into an oral solution, this substance was able to prevent the accumulation of gingival plaque [34], and when incorporated into an adhesive system [15], it was able to inhibit the growth of biofilms, not only of *S. mutans* but also of multispecies. On the contrary, another study [17] did not find an antimicrobial action of composite materials containing nisin (5 and 10%) combined with monocalcium phosphate monohydrate (5 and 10%). A possible explanation for this dissimilarity of results is that those authors did not use the ultrapure version of nisin (nisin A®P) provided by the manufacturer [35]. So, the too low concentration reached in the experimental materials was not able to promote any inhibition for the *S. mutans*, as occurred in the present study, in which pure nisin was used. Indeed, the manufacturer of nisin Z used in that study does not post their MIC (minimal inhibition concentration) for Streptococcus [36], as they do for the ultrapure version.

Similarly, stannous fluoride alone also presented a significant inhibitory effect against *S. mutans*, although its content was not pivotal to this end, like nisin. This result disagrees with the study of Fernández et al. [19], which showed a dose-dependent effect of SnF_2_ on biomass, thickness, and viability of an *S. mutans* biofilm in situ. However, it is important to point out that, in that study, the concentrations of SnF_2_ (15 wt.% and 33 wt.%) were far higher than those used in the present study (0.6, 1.5, and 3 wt.%). Thus, it is reasonable to claim that although these low concentrations were effective in inhibiting the *S. mutans* growth, their nuanced differences were not able to produce different levels of inhibition. When combined, nisin and stannous fluoride (N4SnF_2_0.6) presented more effective inhibition against *S. mutans* than nisin at 4% or 7% alone. This finding aligns with Tong et al. [37], which noted an enhanced antimicrobial effect against *S. mutans* with combined nisin and fluoride. According to these authors, the pores made by nisin in the microorganism’s cell membrane help more fluoride ions enter the cytoplasm to better exert the antibacterial roles of fluoride. On the other hand, it is noteworthy that the inhibitory effect of N4SnF_2_0.6 was significantly lower than that presented by SnF_2_ alone. Nisin is a complex polycyclic peptide with a great number of OH and NH groups in its backbone [38]. Thus, one can hypothesize that this structure could have allowed the establishment of a high level of hydrogen bonds with F from SnF_2_, [39], thereby jeopardizing the entering of fluorides through the *S. mutans* cytoplasm, which can be taken as an antagonistic effect influencing the SnF_2_ inhibitory activity.

A decrease of the demineralization of the surrounding tooth structure is expected from a dental material with antimicrobial potential against *S. mutans,* caused by the production of acids resulting from the microorganism’s sugar metabolism. Concerning stannous fluoride, the presence of fluorides in orthodontic adhesive materials has shown a protective effect against the demineralization of tooth enamel around brackets [7, 40, 41]. Besides, some reports have suggested that the hardness tests provide a sensitive method for evaluating the mineral loss of dental enamel [42, 43]. So, the decrease in enamel hardness around the brackets after the cariogenic challenge found in the present study could be related to the loss of minerals from the tooth structure [42, 43]. It is convenient to note that the indentation for the hardness test was not performed so close to the bracket area to ensure an enamel surface free of composite remnants.

In the present study, a substantial decrease in enamel hardness around the brackets fixed with the composite material without any antimicrobial agent (control group) was observed, which ranged from about 44.5 to 48%, regardless of the distance from the brackets. On the contrary, the decrease in enamel hardness for all the experimental groups containing an antimicrobial agent, regardless of type (nisin or stannous fluoride), concentration, or association, was significantly lower than that of the control group, reflecting a protective effect against tooth demineralization adjacent to bracket edges. However, this protective effect was compromised, but still better than the control group, at a greater distance from the bracket edges (600 µm), except for the N4SnF_2_0.6 group, which showed no significant differences regardless of the distance from the bracket (Table 4). A previous study [41], which investigated an adhesive containing fluoride to fix brackets, also found a lower protective effect for the enamel at greater distances.

The better action of nisin at 4% when associated with stannous fluoride in inhibiting *S. mutans* was also reflected in the greater ability of this group to prevent the reduction of enamel hardness further (600 µm) from the bracket edge. However, the lower antibacterial effect of N4SnF_2_0.6 compared to that of the SnF_2_ groups could not explain its lower reduction of enamel hardness at higher distances. This is a statistical finding that cannot be adequately justified by the authors, thereby impairing any conclusion when comparing the action of these antimicrobial agents. Contrarily, this was not observed at shorter distances. Thus, it can be can be stated that, at lesser distances, all concentrations of the nisin or stannous fluoride, and their combination, might have been enough to cause high inhibition of *S. mutans *and, consequently, to protect the enamel.

The shear bond strength analysis was performed to verify the influence of the addition of nisin and stannous fluoride in composite materials on the adhesive interface. All groups, except for N4SnF_2_0.6, presented shear bond strengths similar to those of the control. This negative effect of the nisin-stannous fluoride association is a finding of the study that, according to the authors’ knowledge, is not found in the literature. However, since a minimum bond strength of around 6 and 8 MPa [44] is suggested for bonding brackets, all groups could be considered satisfactory, since these bond strengths should be considered with caution, as the study was not performed mimicking all oral conditions. Analyzing the failure mode after bracket debonding, score 1 was the most common ARI score found in the present study, indicating a predominance of adhesive failures between the enamel and the composite, result in agreement with previous studies [17, 45]. The ARI score 0 was found only for the N4SnF_2_0.6 group, which could be related to its lower shear bond strength values. In fact, a previous study showed correspondence between higher SBS values and higher ARI scores [46, 47]. The presence of score 0 and 1 is advantageous, as it could avoid enamel damage after bracket debonding, during resin removal procedures.

One limitation of the present study is that only inhibition of *S. mutans* was evaluated, while cariogenic process may involve other pathogenic agents. Another limitation is that the release of nisin and/or SnF_2_ from the composite material to the storage medium was not evaluated. This is a relevant information for predicting the action of these bioactive materials over time. So, future studies can be developed including multispecies biofilm and the release of those bioactive agents, for more accurate assessment of their impact to the enamel protection.

## Conclusion

Our results suggest that the addition of both nisin and stannous fluoride, as well as their combination, can provide an antimicrobial effect against *S. mutans*. This effect offers protective benefits for the dental enamel around brackets without compromising most of the physical properties studied. However, the highest concentration of nisin (10%) and the nisin-stannous fluoride combination were harmful to the degree of conversion and bond strength, respectively.

Notably, SnF_2_ showed remarkable performance, which did not compromise the DC% and led to the highest inhibition, even at the lowest concentration. This suggests the potential for further studies using this combination for the development of antimicrobial dental resin-based products. Future studies should also investigate other mechanical properties and the longevity of the antimicrobial effect of these materials in a challenging oral environment. The antimicrobial potential of dental materials against cariogenic microorganisms can offer substantial benefits for dental caries prevention. Continued investigations in this field can lay the scientific groundwork for the development of commercial materials aimed at improving oral health.

## Data Availability

The data that support the findings of this study are available from the corresponding author upon reasonable request.
